# Research on the visual image-based complexity perception method of autonomous navigation scenes for unmanned surface vehicles

**DOI:** 10.1038/s41598-022-14355-y

**Published:** 2022-06-20

**Authors:** Binghua Shi, Jia Guo, Chen Wang, Yixin Su, Yi Di, Mahmoud S. AbouOmar

**Affiliations:** 1 Hubei University Of Economics, Information and Communication Engineering, Wuhan, 430000 China; 2 No. 722 Research Institute of CSSC, Wuhan, 430000 China; 3 Wuhan University of Technology, School of Automation, Wuhan, 430000 China; 4 Industrial Electronics and Control Engineering Department, Faculty of Electronic Engineering, Menoufia University, 32952 Menouf, Egypt

**Keywords:** Ocean sciences, Engineering

## Abstract

To solve the long-tail problem and improve the testing efficiency for autonomous navigation systems of unmanned surface vehicles (USVs), a visual image-based navigation scene complexity perception method is proposed. In this paper, we intend to accurately construct a mathematical model between navigation scene complexity and visual features from the analysis and processing of image textures. First, the typical complex elements are summarized, and the navigation scenes are divided into four levels according to whether they contain these typical elements. Second, the textural features are extracted using the gray level cogeneration matrix (GLCM) and Tamura coarseness, which are applied to construct the feature vectors of the navigation scenes. Furthermore, a novel paired bare bone particle swarm clustering (PBBPSC) method is proposed to classify the levels of complexity, and the exact value of the navigation scene complexity is calculated using the clustering result and an interval mapping method. By comparing different methods on the classical and self-collected datasets, the experimental results show that our proposed complexity perception method can not only better describe the level of complexity of navigation scenes but also obtain more accurate complexity values.

## Introduction

As various new technologies related to autonomous navigation ships have emerged recently, the main development direction for ships is to be human-free, smart, and intelligent^1^. Some advanced environmental perception, path planning, and motion control methods assist the development of unmanned surface vehicles (USVs)^2–4^. However, to realize accurate remote automatic or autonomous navigation of USVs, a large amount of practical testing is also needed to verify the safety and reliability of their navigation systems^5^. If a USV directly conducts remote autonomous and unmanned driving test experiments in public waters, it will not only be extremely inefficient and costly but will create greater safety problems for themselves and their surrounding navigable vessels^6^. Therefore, it is necessary to assess various navigation scenarios for a USV before conducting real driving test experiments.

Navigation scenario construction technology based on visual sensors has the characteristics of low cost, mature solutions and a high degree of scene restoration, which makes this technology widely used to reconstruct ship navigation scenes in a comprehensive and multiview way^7–9^. Vision-based navigation scene perception provides visual semantic information to human beings that approximate eyesight, which makes this method likely to be widely used in the field of autonomous navigation^10,11^. The greatest challenge in using visual images to construct navigation scenarios is that it is difficult to cover navigation scenes with a small probability of occurrence but high risk, i.e., the “long-tail problem”^12^. Usually, to essentially solve the long-tail problem of scenario coverage, it is necessary to improve the diversity and complexity of the constructed scenarios, and diversity can be obtained by artificially selecting different test conditions. Generally, one of the feasible approaches to address the long-tail problem is to increase the diversity and complexity of navigation scenes^13^. The diversity can be increased by artificially selecting different test conditions. However, the complexity of the navigation scene is a relatively subjective feeling, and a unified perception standard is urgently needed. Furthermore, the perceived complexity of an autonomous driving test system can reflect the difficulty of the construction scenes and the good or poor ability of USVs.

The presentation of navigation scenes based on visual sensors is accomplished through continuous image sequences or videos. The complexity perception method can be designed by referring to the related works in the image engineering field. The main idea of these works is to let a computer simulate human visual perception to make quantitative decisions on the perceived visual complexity of images. The common complexity perception approaches include the following: information entropy^14^, the average information gain^15^, and the compression rate of significant regions^16^. Other methods include quantitative perception methods using image features, such as grayscale, color, edge, and texture^17^, and then using machine learning, neural networks, and other classification regression methods to obtain the complexity perception results. Among the features, textural features are one of the most commonly used feature objects in image content complexity calculations. For example, Guo et al.^18^ used the textural features of regularity, orientation, density, roughness, and familiarity to describe image complexity. Chen et al.^19^ applied BP neural networks to train five index weight coefficients, including the energy, entropy, contrast, homogeneity, and correlation and then built an image complexity perception model. The experimental results showed that their proposed model can accurately describe the complexity of images. Zhan et al.^20^ proposed an autonomous visual perception method for unmanned surface vehicle navigation based on the local color and texture features. Experiments in lakes with various scenes demonstrate that the proposed method can recognize unknown environments. To assess the complexity of an image, machine learning has two main categories of methods: regression and classification^21^. The regression-based methods include linear or nonlinear fitting^22^ and random forest fitting algorithms^23^, which have the disadvantage of being prone to overfitting when the size of the dataset is small. The classification-based methods include support vector machines (SVMs)^24,25^, BP neural networks^21^, and convolutional neural networks (CNNs)^26,27^, but these methods are also more demanding on datasets and require manual labeling. Different from the above methods, unsupervised clustering-based methods for image complexity perception have also been proposed^28^. These methods use K-means or particle swarm optimization (PSO) algorithms to cluster data samples with the same characteristics together for the purpose of complexity assessment.

The above approaches in the image engineering field tend to qualitatively describe images using the overall image complexity and lack results focusing on the complexity analysis of USV navigation scenes. In addition, the complexity result is easily affected by various aspects, such as light irradiation, object surface reflection and the internal performance parameters of an imaging sensor. In particular, the complexity of a scene varies greatly under different navigation conditions, which increases the difficulty of calculating and perceiving the complexity of navigation scenes. Hence, this paper considers the special scenarios for USV, analyzes the typical complex elements, proposes the complexity perception method of a navigation scene based on image texture features, optimizes the unsupervised PSO features clustering algorithm and explores the feasibility of our proposed method on classical and self-harvested datasets. The remainder of this paper is organized as follows. Section “Vision-based navigation scene” presents and analyzes some typical complex elements for vision-based navigation scenes. Section “Design of the complexity perception method” proposes our novel algorithms and their implementation, and the results and discussions are given in Sections “Experiments and results” and “Discussion”, respectively. Finally, some conclusions and future research are discussed in Section “Conclusions”.

## Vision-based navigation scene

### Typical complex elements

**Figure 1:**
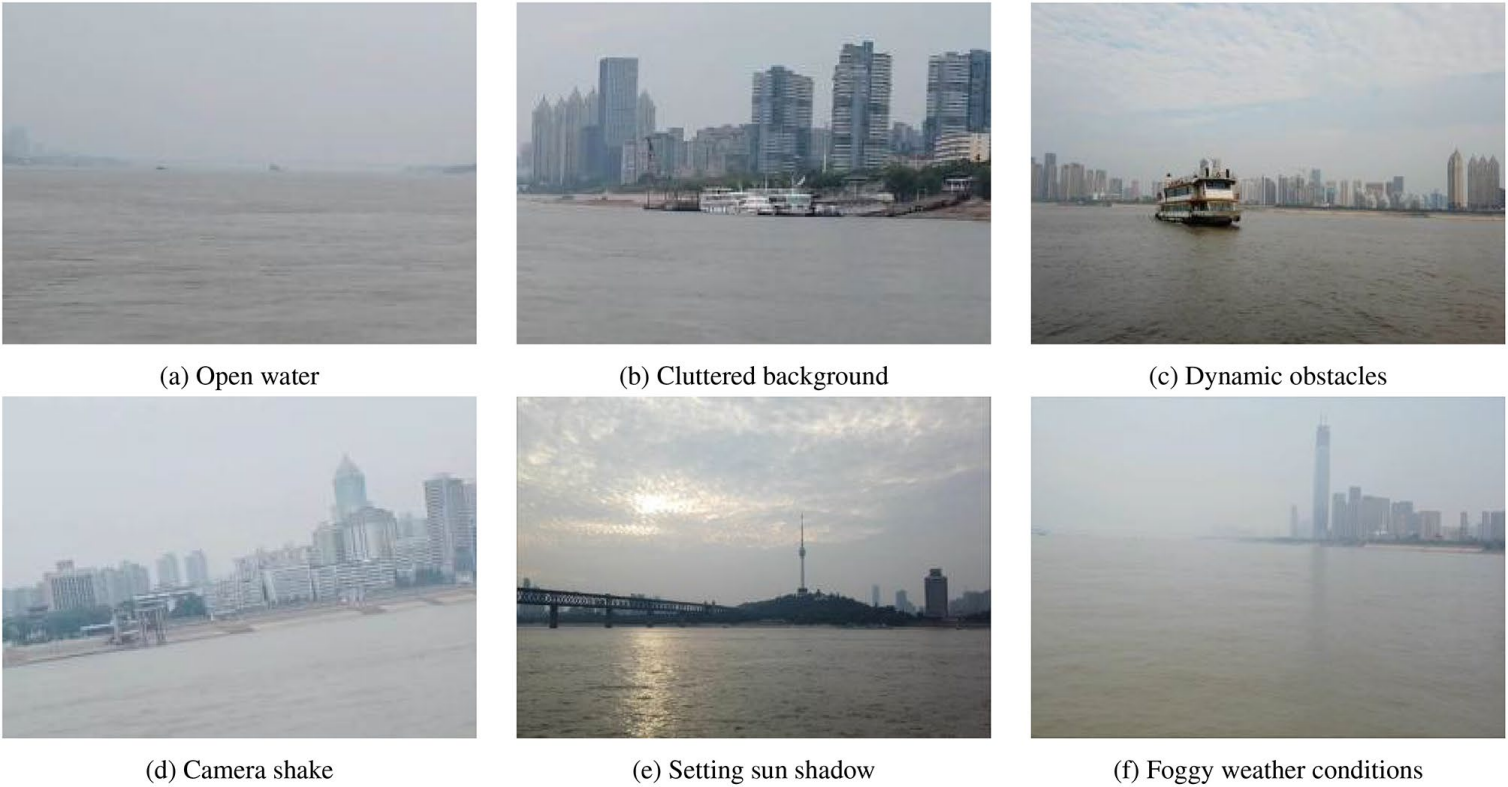
Examples of open water and 5 typical complex elements.

To cover as many test scenarios as possible and solve the long-tail problem when constructing navigation scenarios for USVs, the typical complex elements that need to be focused on are systematically determined. Before a USV is driven for testing, it is necessary to have knowledge of the type of water to be navigated, i.e., to determine whether the navigation scenes are in open water or restricted water. Open water usually refers to a sea with an open view, as shown in Fig. 1a. Conversely, restricted water includes inland rivers or ports with heavy traffic^29^. Typical complex elements usually occur in restricted waters because there are many uncertain features during navigation testing. A classification of these features reveals that a complex navigation scene contains the following typical elements:

- **Cluttered background** A cluttered background will occupy a certain proportion of the navigation scenes, and the pixel values of the corresponding area will change irregularly once the test scenarios include complex elements^30^. For example, distant city buildings and plants on the shore are shown in Fig. 1b. These cluttered backgrounds do nothing but increase the complexity of navigation scenes and interfere with the environmental perception of USVs.
- **Dynamic obstacles**. Affected by the environment or a ship’s movement, navigation scenes contain disturbances caused by dynamic obstacles and a static background. For instance, moving ships (as shown in Fig. 1c), floating obstacles and other traffic participants can increase the risk and make a navigation scene more complex^31^.
- **Camera shaking** Vision-based navigation scenes have to consider the camera shaking phenomenon, which is caused by wind, waves and currents on the water. In addition, the phenomenon is even more serious when a ship is moving^32^. Camera shaking can lead to skewed, rotated or even distorted scene images, adding to the complexity, as shown in Fig. 1d.
- **Cast shadows** The sun, clouds, ships and buildings in a navigation scene can all produce cast shadows, which can be classified as self-shadows and projected shadows. A self-shadow is formed by the part of light being blocked directly, and a projected shadow is formed by an object being projected onto the water surface, i.e., the reflection of the sun setting (as shown in Fig. 1e), clouds, trees and buildings on the shore.
- **Special weather conditions** Rain, snow, night and fog will affect the complexity of a navigation scene. Special weather conditions, such as rain and snow, lead to different scene performances. For example, raindrops and snowflakes are easily captured and thus interfere with the perception of other targets. Night or foggy conditions will lead to a decrease in imaging contrast, blurred images and loss of details. This is shown in Fig. 1f.

### Division of levels of complexity

To determine the complexity of a navigation scene, we judge whether the scene is complex according to whether it contains the typical elements and how many elements are included to divide the levels of complexity. The specific case method is shown in Table 1.

**Table 1:** Description of the levels of complexity.

No.	Abbr.	Levels	Description
Case 1	$L_{N}$	No complexity	Does not contain any typical elements
Case 2	$L_{L}$	Low complexity	Contains any of typical elements
Case 3	$L_{M}$	Medium complexity	Contains any two typical elements
Case 4	$L_{H}$	High complexity	Contains three or more typical elements.

**Figure 2:**
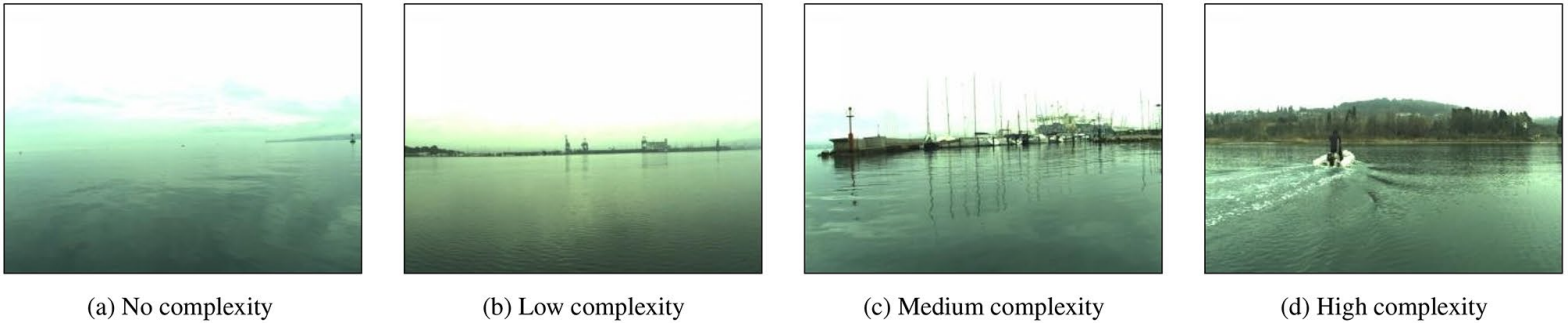
Examples of levels of complexity.

The example images in Fig. 2 are taken from the marine obstacle detection dataset (MODD)^33^. The no-complexity scene in Fig. 2a shows clear weather and an open view, which is basically free of shadows and does not affect the safe navigation of the USV. Fig. 2b contains projected shadow elements on the shoreline that form reflections on the water, causing this navigation scene to appear slightly less complex. Figure 2c contains the cluttered background and cast shadow elements in which the dark areas in the image of the navigation scene are relatively large, showing a medium-complexity scenario. Figure 2d contains not only a cluttered background and cast shadow elements but also dynamic obstacles, and the navigation scene is highly complex under the influence of three typical elements.

## Design of the complexity perception method

### Methodology framework

**Figure 3:**
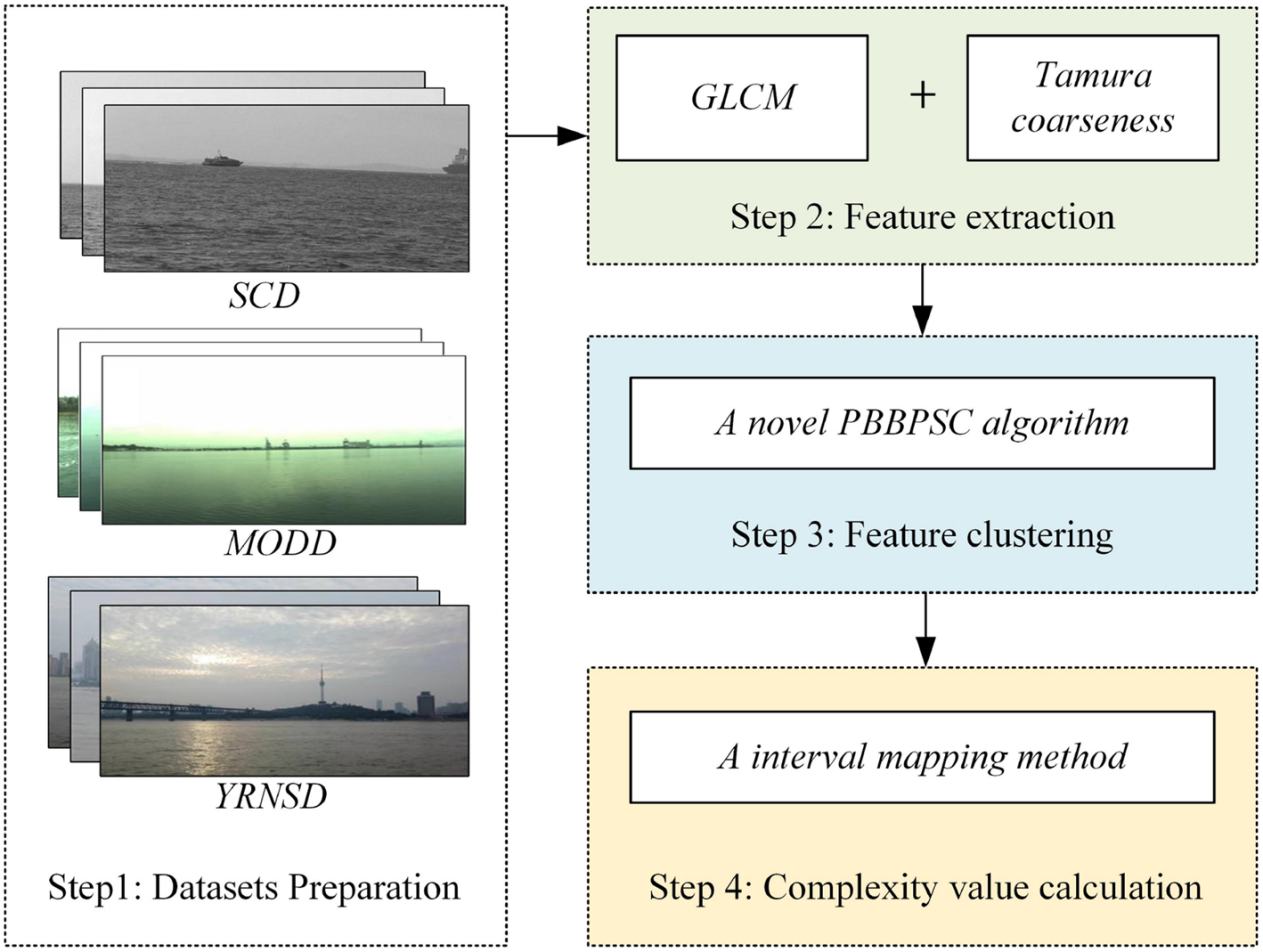
The framework of the complexity perception method.

As the integral link of autonomous navigation testing, the complexity perception of a navigation scene based on a visual sensor relies on feature extraction and processing. The presence of the abovementioned typical complex elements makes the frame images captured by visual sensors exhibit different textural features, and the extraction and clustering of these features are closely related to the results of the complex perception of the navigation scene. The complexity perception results are reflected in two aspects: level determination and complexity value calculation. As shown in Fig. 3, our complexity perception of the navigation scene includes the following steps: (1) dataset preparation, (2) feature extraction, (3) feature clustering, and (4) complexity value calculation. The specific implementation process of each step is as follows.

### Texture feature parameter extraction

Image texture information is one of the important bases to reflect whether a navigation scene is complex. Referring to the related results in the field of image complexity analysis, the textural features can be obtained using the gray level cogeneration matrix (GLCM) and Tamura methods. By computing the GLCM of an image to obtain its feature information, Haralick^34^ proposed 14 statistical feature parameters, including the energy, entropy, contrast, homogeneity, correlation, and variance. Tamura’s method is based on the human visual perception of texture, and Hideyuki Tamura^35^ approximated six basic textural features in computational form: coarseness, contrast, directionality, line-likeness, regularity, and roughness. Combining the above methods results in 20 feature parameters describing the complexity of a navigation scene from different aspects, but there is an overlap problem and redundancy among these feature parameters. Therefore, this paper selects five textural parameters with low correlation that are easy to calculate, including the energy, contrast, inverse difference moment, correlation, and roughness, for the complexity perception of a navigation scene.

#### Feature parameters based on a GLCM

Assume an $M \times N$ navigation scene *I* with $N_g$ gray grades, $\left( x_{1}, y_{1}\right)$ and $\left( x_{2}, y_{2}\right)$ are two pixel points in scene *I* with distance *d* in the direction of $\theta$. Then, the GLCM of this navigation scene is calculated as follows:

1 $$\begin{aligned} \begin{aligned} P(i, j, d, \theta )=\#\{\left( x_{1}, y_{1}\right) ,\left( x_{2}, y_{2}\right) \in M \times N \mid I\left( x_{1}, y_{1}\right) =i, I\left( x_{2}, y_{2}\right) =j \} \end{aligned} \end{aligned}$$

where $\#$ denotes the number of elements in the set. $i,j=0,1,2,\ldots ,N_g-1$ represents the gray levels of two pixels.

The energy (*ASM*) is used to describe the uniformity of the distribution of the navigation scene. When the elements are concentrated near the diagonal of the GLCM, a smaller value of *ASM* indicates that the grayscale distribution is more uniform and the texture is finer; conversely, it indicates that the grayscale distribution is uneven and the texture is rougher.

2 $$\begin{aligned} A S M=\sum _{i=0}^{N_{g}-1} \sum _{j=0}^{N_{g}-1} P(i, j, d, \theta )^{2} \end{aligned}$$

The contrast (*CON*) is used to reflect the depth of image texture grooves and clarity. In a particular navigation scene, a clearer image texture means a larger value of *CON*, and the opposite means a smaller value.

3 $$\begin{aligned} C O N=\sum _{i=0}^{N_{g}-1} \sum _{j=0}^{N_{g}-1}(i-j)^{2} P(i, j, d, \theta ) \end{aligned}$$

The inverse difference moment (*IDM*) is a statistical feature parameter that reflects the local texture of a navigation scene. When the value of *IDM* is large, it indicates that the textures of different regions in this navigation scene are more homogeneous.

4 $$\begin{aligned} I D M=\sum _{i=0}^{N_{g}-1} \sum _{j=0}^{N_{g}-1} \frac{P(i, j, d, \theta )}{1+(i-j)^{2}} \end{aligned}$$

The correlation (*COR*) is used to measure the similarity of the GLCM elements in the row or column direction. When the row or column similarity is high, the value of *COR* is larger, and the complexity of the scene is smaller. The opposite also holds.

5 $$\begin{aligned} C O R=\sum _{i=0}^{N_{g}-1} \sum _{j=0}^{N_{g}-1}\left( i-\mu _{1}\right) \left( j-\mu _{2}\right) / \delta _{1} \delta _{2} \end{aligned}$$

where $\mu _{1}$ and $\mu _{2}$ denote the mean values of the elements along the normalized GLCM in the row and column directions, respectively. $\delta _{1}$ and $\delta _{2}$ represent their mean squared values.

#### Feature parameter based on Tamura coarseness

**Figure 4:**
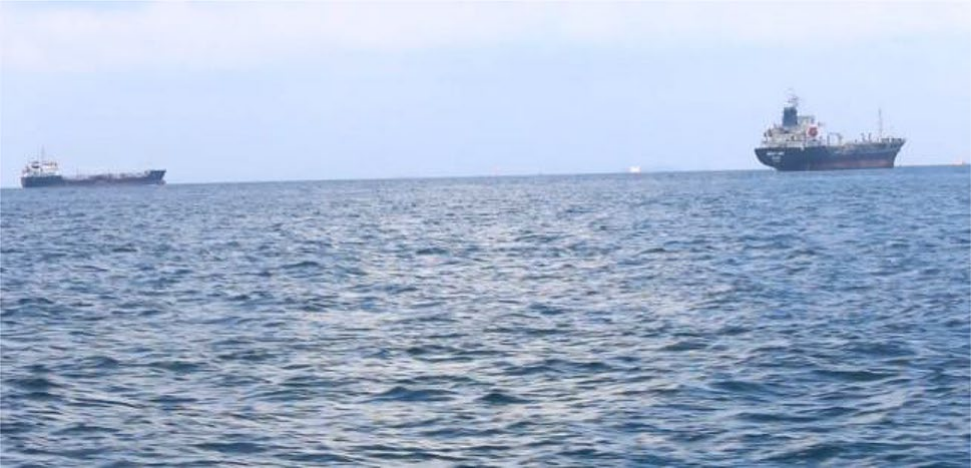
Coarseness of water surface.

The coarseness (*COA*) is related to the distances of notable spatial variations of gray levels. That is, the coarseness is implicitly related to the size of the primitive elements forming the texture of the navigation scene. Since the water surface is easily rippled by wind and wave currents and other meteorological environments, wave texture is one of the elements that cannot be ignored in ship navigation scenes, as shown in Fig. 4 (referring to the classical SMD dataset^13^). The wave texture on the water surface is closely related to the complexity of the navigation scenes, and the intensity of the wave texture can be characterized by the Tamura coarseness. Therefore, when the value of *COA* is larger, the water surface is not calm, and the complexity of the navigation scene is high.

For an $M \times N$ navigation scene *I*, the calculation of coarseness of the navigation scene starts by selecting a $2^{k} \times 2^{k}$ pixel sliding window and obtaining the mean value of the pixel intensity $A_{k}(x, y)$ after traversing the sliding window:

6 $$\begin{aligned} A_{k}(x, y)=\sum _{m=x-2^{k-1}}^{x+2^{k-1}-1} \sum _{n=y-2^{k-1}}^{y+2^{k-1}-1} I\left( m, n \right) / 2^{2 k} \end{aligned}$$

where $k=1,2, \ldots , K$ controls the size of the sliding window, and *K* generally takes values between 2 and 6. (*x*, *y*) denotes the pixel points within the sliding window. $I\left( m, n \right)$ is the gray level at point (*m*, *n*).

Then, the average intensity difference between windows that do not overlap with each other is calculated by:

7 $$\begin{aligned}&D_{k, h}(x, y)=\left| A_{k}\left( x+2^{k-1}, y\right) -A_{k}\left( x-2^{k-1}, y\right) \right| \end{aligned}$$

8 $$\begin{aligned}&D_{k, v}(x, y)=\left| A_{k}\left( x, y+2^{k-1}\right) -A_{k}\left( x, y-2^{k-1}\right) \right| \end{aligned}$$

where $D_{k, h}(x, y)$ denotes the intensity difference in the horizontal direction, and $D_{k, v}(x, y)$ denotes the intensity difference in the vertical direction. For each pixel, assuming that $k_{best }$ is the value of *k* that maximizes the larger of $D_{k, h}(x, y)$ and $D_{k, v}(x, y)$, then the corresponding optimal size $S_{\text{ best } }\left( m, n\right)$ is $2^{k_{best }}$ at this time. The average of the optimal sizes is calculated as follows:

9 $$\begin{aligned} C O A=\frac{1}{M \times N} \sum _{m=1}^{M} \sum _{n=1}^{N} S_{b e s t}\left( m, n\right) \end{aligned}$$

Therefore, for any USV navigation scene, we extract five parameters separately and combine them into a texture feature vector as follows:

10 $$\begin{aligned} \mathbf{E }=[A S M, C O N, I D M, C O R, C O A] \end{aligned}$$

### Feature clustering based on the PBBPSC algorithm

To better perceive the complexity of a navigation scene, it is necessary to obtain 4 complexity-level texture feature clustering centers $\hat{\mathbf{L }}=\left\{ {\hat{L}}_{N}, {\hat{L}}_{L}, {\hat{L}}_{M}, {\hat{L}}_{H}\right\}$ using a paired bare bones particle swarm clustering (PBBPSC) approach, which is modified from the classical and unsupervised particle swarm optimization (PSO) algorithm. The objective function of the feature clustering problem is:

11 $$\begin{aligned} f_{obj} :~~~ min\left\{ S_{dis}(\mathbf{E },\hat{\mathbf{L }})\right\} \end{aligned}$$

$S_{dis}(\mathbf{E },\hat{\mathbf{L }})$ denotes the sum distance of the feature particles from the clustering centers.

12 $$\begin{aligned} S_{dis}(\mathbf{E },\hat{\mathbf{L }})=\sum _{i=1}^{N_{\mathbf{E }}}\sum _{j=1}^{4} {{\varvec{M}}}_{ij}{{\varvec{D}}}_{ij} \end{aligned}$$

where $N_{\mathbf{E }}$ represents the length of the texture feature vector $\mathbf{E }$. In the above equations, ${{\varvec{D}}}$ is the distance matrix, and ${{\varvec{D}}}_{ij}$ denotes the distance between the *i*th vector data and the *j*th cluster center. Furthermore, ${{\varvec{M}}}$ is the belonging matrix, and ${{\varvec{M}}}_{ij}$ is expressed as:

13 $$\begin{aligned} {{\varvec{M}}}_{ij}=\left\{ \begin{array}{ll} 1 &{} if \quad {{\varvec{D}}}_{ij}=min\left\{ \textit{D}_{i1}, \textit{D}_{i2},\textit{D}_{i3},\textit{D}_{i4}\right\} \\ 0 &{} else \quad other \end{array}\right. \end{aligned}$$

The purpose of our clustering algorithm is to find $\hat{\mathbf{L }}$ that can minimize the objective function. Therefore, a sample and effective PBBPSC approach is used to investigate the best position of the cluster centers. At the beginning of clustering, initial positions for all particles are generated randomly, and then the first positions and personal best values can be obtained. During the iterative process, a paired operator (PO) is applied to cross local minimums. The PO forms particles to search in pairs. In each pair, the particle with a small personal best value is the main particle, and the other particle is the side particle. The candidate position of the main particle is calculated by:

14 $$\begin{aligned}&\alpha =\frac{(P_{best\_main}^t+G_{best}^t)}{2} \end{aligned}$$

15 $$\begin{aligned}&\beta =|P_{best\_main}^{t}-G_{best}^t| \end{aligned}$$

16 $$\begin{aligned}&P_{cand\_main}^{t+1}=Gauss(\alpha ,\beta ) \end{aligned}$$

where $P_{best\_main}$ is the personal best position of the main particle, $G_{best}$ is the global best position of the swarm, $P_{cand\_main}$ is the candidate position of the main particle, and $Gauss(\alpha ,\beta )$ is the Gaussian distribution with mean $\alpha$ and standard deviation $\beta$.

Similarly, the candidate position of a side particle is calculated by:

17 $$\begin{aligned}&\gamma =\frac{(P_{best\_main}^t+P_{best\_side}^t)}{2} \end{aligned}$$

18 $$\begin{aligned}&\delta =|P_{best\_main}^{t}-P_{best\_side}^t| \end{aligned}$$

19 $$\begin{aligned}&P_{cand\_side}^{t+1}=Gauss(\gamma ,\delta ) \end{aligned}$$

where $P_{best\_side}$ and $P_{cand\_side}$ are the personal best and candidate positions of the side particle, respectively. $Gauss(\gamma ,\delta )$ is the Gaussian distribution with mean $\gamma$ and standard deviation $\delta$.

In each iteration, the personal best positions and values and the global best position and value can be updated after all particles have calculated their candidate positions. Once the number of iterations reaches the preset value, the final global best value and the position are the solutions presented by the PBBPSC approach. The pseudocode of PBBPSC is shown in Algorithm  1.

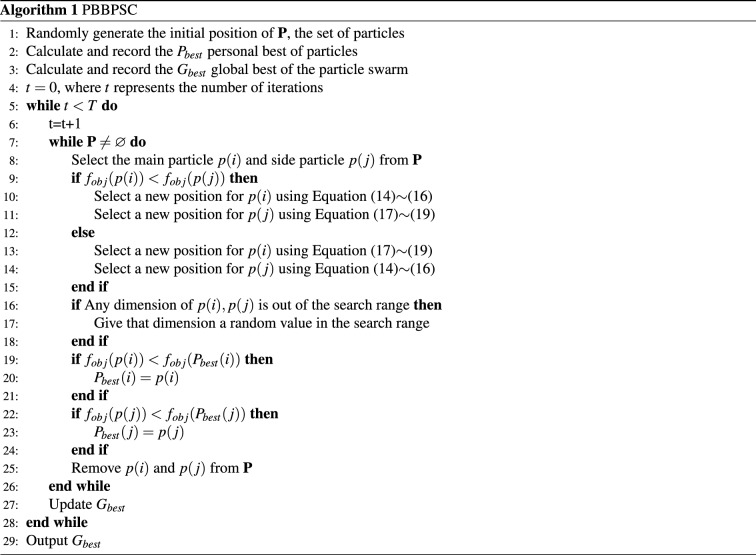



### Complexity calculation based on the interval mapping method

The level of complexity of a navigation scene can be obtained by the above clustering method, and we use a sample interval mapping function to calculate the complexity value as follows:

**Step 1:** Compare the Euclidean distance. Calculate the distance *d*(*E*, *L*) between the textural features of scene *E* and the cluster centers at each level of complexity.

20 $$\begin{aligned} d(E, L)=|E-L|=\sqrt{\sum _{k=1}^{5}(E(k)-L(k))^{2}} \end{aligned}$$

**Step 2:** Determine the levels of complexity. If the distance from navigation scene $E_{t}$ to the cluster centers of the four classes ${\hat{L}}_{N}, {\hat{L}}_{L}, {\hat{L}}_{M}, {\hat{L}}_{H}$ satisfies the following equation, the level of complexity of this scene is $L_{j}$:

21 $$\begin{aligned} d\left( E_{t}, {\hat{L}}_{j}\right) <d\left( E_{t}, {\hat{L}}_{i}\right) , \quad i \ne j \mid i, j=\{N, L, M, H\} \end{aligned}$$

**Step 3:** The value of the complexity is distinguished. Map the four levels $\psi \left( L_{N}\right) , \psi \left( L_{L}\right) , \psi \left( L_{M}\right) , \psi \left( L_{H}\right)$ to the interval [0, 1], i.e., the range of complexity values corresponds to the following equation:

22 $$\begin{aligned} \left\{ \begin{array}{l} \psi \left( L_{N}\right) \in [0,0.25) \\ \psi \left( L_{L}\right) \in [0.25,0.5) \\ \psi \left( L_{M}\right) \in [0.5,0.75) \\ \psi \left( L_{H}\right) \in [0.75,1] \end{array}\right. \end{aligned}$$

when the navigation scene $E_{t}$ is classified as $L_{j}$ and if $d\left( E_{t}, {\hat{L}}_{j}\right) =0$, then $\psi \left( {\hat{L}}_{j}\right) \in \{0.125,0.375,0.625,0.875\}$

**Step 4:** Calculate the final complexity value. The cluster center of each level of complexity ${\hat{L}}_{j}$ divides that complexity interval into the left part $\left| E_{t}\right| <\left| {\hat{L}}_{j}\right|$ and the right part $\left| E_{t}\right| >\left| {\hat{L}}_{j}\right|$ in turn, and the complexity value corresponding to each part is calculated as follows:

23 $$\begin{aligned} \psi \left( E_{t}\right) =\left\{ \begin{array}{ll} \psi \left( {\hat{L}}_{j}\right) &{} \left| E_{t}\right| =\left| {\hat{L}}_{j}\right| \\ max \left\{ \psi \left( {\hat{L}}_{j}\right) -0.125,\psi \left( {\tilde{E}}_{t}\right) \right\} &{} \left| E_{t}\right| <\left| {\hat{L}}_{j}\right| \\ min \left\{ \psi \left( {\tilde{E}}_{t}\right) ,\psi \left( {\hat{L}}_{j}\right) +0.125\right\} &{} \left| E_{t}\right| >\left| {\hat{L}}_{j}\right| \end{array}\right. \end{aligned}$$

Furthermore, $\psi \left( {\tilde{E}}_{t}\right)$ can be obtained by the equation:

24 $$\begin{aligned} \psi \left( {\tilde{E}}_{t}\right) =\psi \left( {\hat{L}}_{j}\right) +\frac{1}{8}\frac{d\left( E_{t}, {\hat{L}}_{j}\right) -\mu _{d\left( E_{t}, {\hat{L}}_{j}\right) }}{\sigma _{d\left( E_{t}, {\hat{L}}_{j}\right) }} \end{aligned}$$

where $\mu _{d\left( E_{t}, {\hat{L}}_{j}\right) }$ is the average distance from the scene complexity samples to the respective hierarchical clustering centers, and $\sigma _{d\left( E_{t}, {\hat{L}}_{j}\right) }$ is the standard variance in the distance.

## Experiments and results

### Datasets

To enrich the diversity of the testing scenarios, the effectiveness of the proposed method is verified on the classical Singapore marine dataset (SMD), MODD, and our self-collected Yangtze River navigation scene dataset (YRNSD)^36^. The classical SMD contains navigation scenes in various environmental conditions obtained using a Canon 70D camera in waters around Singapore from July 2015 to May 2016^13^. The classical MODD comes from multiple platforms, most of which were small unmanned surface vehicles (USVs), which were manually operated in the port of Koper, Slovenia over a period of months at different times of the day under different weather conditions^33^. Our YRNSD dataset contains a total of 64 videos recorded in the Wuhan section of the Yangtze River and covers many types of obstacles and meteorological conditions. The complexity of the datasets was annotated using manual classification based on whether the frame images contain typical complex elements and the number of their types. The corresponding annotated results are shown in Table 2.

**Table 2:** Datasets and their levels of complexity (unit: frame).

Datasets	No complexity	Low complexity	Medium complexity	High complexity	Size of frames (:pixels)
SMD	30	856	2242	1132	1920 × 1080
MODD	378	3213	806	687	640 × 480
YRNSD	583	1672	2770	1443	1920 × 1080

### Experimental setup

**Figure 5:**
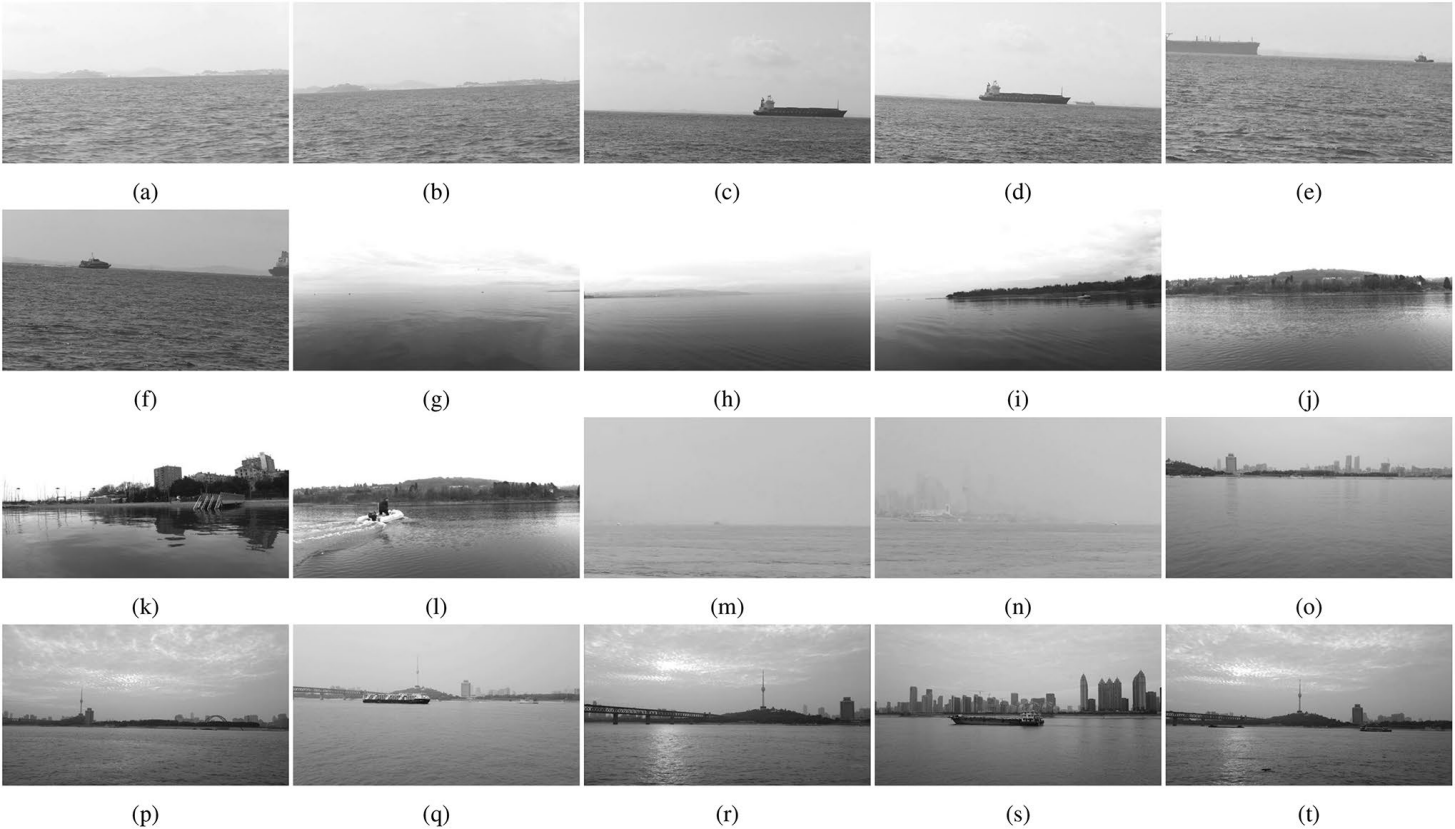
Random navigation scenes from the testing set.

All experiments are implemented on a personal computer with an Intel Core i7-8700K @ 3.70 GHz*12 CPU, an NVIDIA GeForce GTX 1080Ti GPU, 32 GB RAM, and the 64-bit Windows 7 operating system. To ensure testing efficiency and save computational resources, the sizes of the frames in the above datasets are unified to 500 × 280 before clustering and calculation. Then, a 5-dimensional parameter vector can be obtained after textural feature extraction. The above datasets are divided into training and testing sets at rates of 70% and 30%, respectively. Figure 5 shows some randomly selected scenes in the testing set, and their corresponding textural feature parameters are shown in Table 3.

**Table 3:** Test images with different complexities.

Datasets		*ASM*	*CON*	*IDM*	*COR*	*COA*
SMD	Figure 5a	0.0595	0.9881	0.1773	0.9708	0.7725
	Figure 5b	0.0078	0.0991	0.2302	0.9457	0.7345
	Figure 5c	0.9029	0.3379	0.0286	0.0645	0.9139
	Figure 5d	0.9373	0.3067	0.0298	0.0350	0.9025
	Figure 5e	0.0571	0.9877	0.4210	0.9727	0.0262
	Figure 5f	0.0472	0.9845	0.2573	0.9801	0.2297
MODD	Figure 5g	0.0607	0.9300	0.5461	0.9082	0.2044
	Figure 5h	0.7380	0.1781	0.3543	0.1696	0.5658
	Figure 5i	0.6774	0.4270	0.3459	0.3795	0.5371
	Figure 5j	0.9263	0.3205	0.0174	0.0436	0.9945
	Figure 5k	0.2479	0.7603	0.4776	0.7667	0.2792
	Figure 5l	0.2473	0.7704	0.3639	0.7486	0.3769
YRNSD	Figure 5m	0.0793	0.9860	0.2864	0.3618	0.1563
	Figure 5n	0.5583	0.4454	0.2654	0.3895	0.6688
	Figure 5o	0.5095	0.4674	0.5619	0.4893	0.4722
	Figure 5p	0.9379	0.3177	0.0042	0.0291	0.9703
	Figure 5q	0.9516	0.2828	0.0213	0.0362	0.9077
	Figure 5r	0.0653	0.9812	0.2904	0.9674	0.2395
	Figure 5s	0.0263	0.9721	0.3772	0.9869	0.1495
	Figure 5t	0.0205	0.9771	0.4054	0.9897	0.0390

### Results comparison and analysis

To verify and compare the effectiveness of our proposed method, we conduct a comparative analysis of the feature clustering and the calculated values of the levels of complexity of the navigation scenes.

#### Comparison of different clustering methods

The level of complexity of each navigation scene of the training set is manually graded in advance based on its inclusion of typical elements. The manual grading results are used as references, and our proposed PBBPSC approach clustering results are used as the actual results. To evaluate the effectiveness of our proposed approach, it is compared with the K-means algorithm and the classical particle swarm clustering (PSC) algorithm using a series of mathematical parameters, such as the *purity*, *precision*, Rand index (*RI*), and adjusted Rand index (*ARI*). *ARI* is an improved version of *RI* that aims to remove the random effects on the results. These mathematical parameters are calculated as follows:

25 $$\begin{aligned} Purity=\sum _{i=1}^{4}\frac{m_i}{m}P_i \end{aligned}$$

*m* denotes the total number of testing navigation scenes, and $m_i$ is the number of all members in cluster *i*. $P_i$ represents the max value of $P_{ij}$, which refers to the probability that a member of cluster *i* belongs to class *j*.

26 $$\begin{aligned}&Precision=\frac{TP}{TP+FP} \end{aligned}$$

27 $$\begin{aligned}&RI=\frac{TP+TN}{TP+FN+FP+TN} \end{aligned}$$

28 $$\begin{aligned}&ARI=\frac{2 \times (TP\cdot TN-FN\cdot FP )}{(TP+FN)(FN+TN)+(TP+FP)(FP+TN)} \end{aligned}$$

Among the components, *TP* represents the number of navigation scenes where two samples with the same level of complexity are in the same cluster. *FP* represents the number of navigation scenes where two samples with different levels of complexity are in the same cluster. *TN* denotes the number of scenes in which two samples with different levels of complexity are in two separate clusters. *FN* denotes the number of scenes in which two similar levels of complexity are in two separate clusters.

The above mathematical parameters all have values in the interval [0, 1], and the larger the value is, the better. Table 4 shows the clustering results of these three methods.

**Table 4:** Evaluation parameters of the three clustering methods.

Methods	*Purity*	*Precision*	*RI*	*ARI*	Running time (unit:s)
K-means	0.8595	0.7674	0.8254	0.5851	**1.3380**
PSC	0.9273	0.9020	0.8974	0.7424	5.5780
PBBPSC	**0.9782**	**0.9619**	**0.9549**	**0.8828**	6.2680

Significant values are in bold.

**Figure 6:**
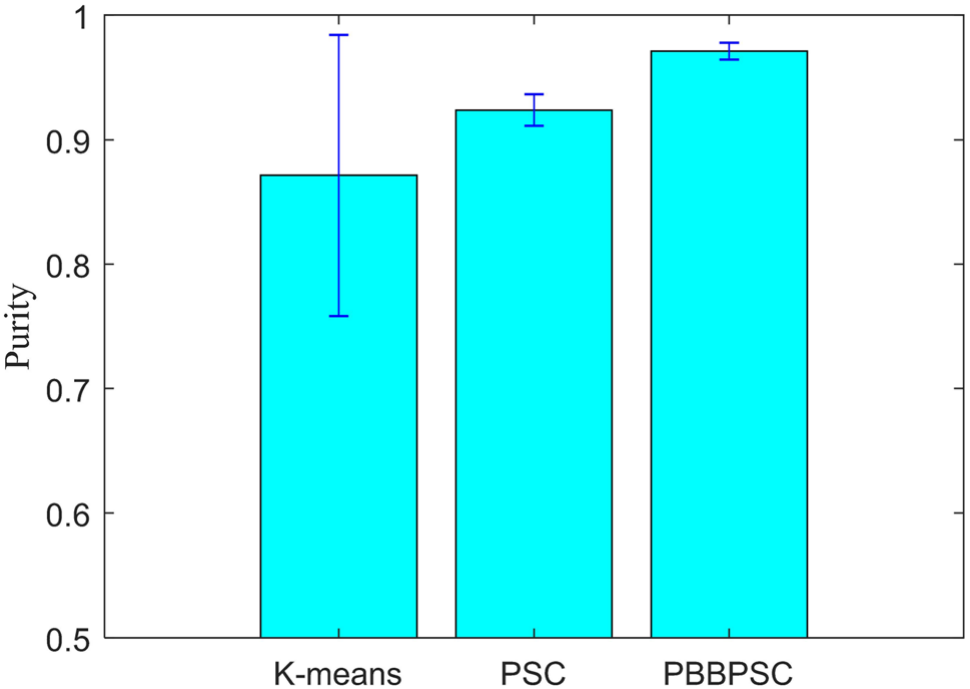
Stability of the three clustering methods.

The results shown in Table 4 show that the novel proposed PBBPSC clustering method achieves the best performance on the above evaluation parameters in addition to the running time. However, the time complexity of all three methods is *O*(*n*) in theory. In addition, to further verify the stability of the PBBPSC clustering method, the above experiment was repeated 30 times, and the stability effect of each clustering method is shown in Fig. 6. The results demonstrate that the PBBPSC clustering method has the best mean and standard deviation over 30 independent runs, which indicates the better stability of our proposed method.

#### Comparison of different complexity calculation methods

The complexity value of a navigation scene can be precisely calculated according to the extracted textural features. First, specific image feature parameters are usually selected as evaluation metrics, and the selected evaluation metrics are normalized and transformed into metrics that are positively correlated with the image complexity. Then, the average weighting method^37^ or BP neural network^19^ is used to obtain the weight coefficients of each evaluation metric, and the final step multiplies the evaluation metrics with the weights to obtain the complexity value. In this paper, the energy, contrast, inverse difference moment, correlation, and roughness are used as evaluation indexes, and the average weighting method (AWM), BP neural network weighting method (BPNNWM), and our proposed method are used to calculate the complexity. The specific results are shown in Table 5.

**Table 5:** Test images of different complexity.

Sample data	Levels	Reference intervals	AWM	BPNNWM	Our method
Figure 5a	$$ L_N$$	[0, 0.25)	0.5963	0.2641	**0.2395**
Figure 5b	$$ L_L$$	[0.25, 0.5)	**0.4035**	**0.3688**	**0.2570**
Figure 5c	$$ L_M$$	[0.5, 0.75)	0.4496	**0.7727**	**0.6262**
Figure 5d	$$ L_M$$	[0.5, 0.75)	0.4423	**0.6618**	**0.6563**
Figure 5e	$$ L_H$$	[0.75, 1]	0.4928	**0.9515**	**0.7919**
Figure 5f	$$ L_H$$	[0.75, 1]	0.4998	** 0.9536**	**0.9422**
Figure 5g	$$ L_N$$	[0, 0.25)	0.5304	0.2957	**0.1345**
Figure 5h	$$ L_N$$	[0, 0.25)	**0.4012**	**0.1956**	**0.1824**
Figure 5i	$$ L_L$$	[0.25, 0.5)	**0.4734**	**0.3888**	**0.4416**
Figure 5j	$$ L_M$$	[0.5, 0.75)	0.4605	0.7687	**0.7772**
Figure 5k	$$ L_H$$	[0.75, 1]	0.5065	**0.9598**	**0.9943**
Figure 5l	$$ L_H$$	[0.75, 1]	0.5014	**0.9648**	**0.9180**
Figure 5m	$$ L_N$$	[0, 0.25)	0.3740	**0.0897**	**0.1390**
Figure 5n	$$ L_L$$	[0.25, 0.5)	**0.4715**	0.1869	**0.1495**
Figure 5o	$$ L_L$$	[0.25, 0.5)	0.5000	**0.4841**	**0.4297**
Figure 5p	$$ L_M$$	[0.5, 0.75)	0.4518	**0.6708**	**0.6725**
Figure 5q	$$ L_M$$	[0.5, 0.75)	0.4399	**0.6801**	**0.7297**
Figure 5r	$$ L_H$$	[0.75, 1]	0.5088	**0.9616**	**0.8595**
Figure 5s	$$ L_H$$	[0.75, 1]	0.5024	**0.9663**	**0.9014**
Figure 5t	$$ L_H$$	[0.75, 1]	0.4863	**0.9640**	**0.9117**

The values in bold in Table 5 indicate that the complexity values are within the reference intervals, which means that the scene is validly perceived. The complexity values obtained by the AWM are prone to mismatches between the calculated value and the actual level. For example, the level of complexity of the navigation scene in Fig. 5a is $$ L_N$$, but its complexity value is greater than that of the reference interval with a high level of complexity, and this result is obviously improper. The BPNNWM method largely improves the shortcomings of the AWM, but it easily falls into the local optimal solution, and each training initial value and process has a slight difference that may lead to a large difference in the complexity value of the same navigation scene; that is, there is randomness in the calculation results. Table 5 shows that the complexity values calculated by our proposed method can match the reference intervals well. First, this is because the texture parameter features are relatively fixed. Second, the accuracy of the proposed clustering results can be maintained at a high level. Therefore, the complexity values computed by interval mapping can possess good stability during the complexity perception process.

## Discussion

During the USV tests, we concluded that the actual distribution of the four levels of complexity scenarios deviated from the distribution of the actual dataset used in this paper. Both the classical and self-harvested datasets in our experiments focus on scenarios with high complexity and smaller proportions of no-complexity scenes. Taking the self-collected YRNSD as an example, the Wuhan section of the Yangtze River basin is typical inland river water, and the characteristics of the collected frames are characterized by busy traffic, complex backgrounds on both sides of the river, and narrow channels. Therefore, the proportion of no-complexity scenes is very small in the data samples. To balance the data samples, we performed the data augmentation operations of rotation, translation, scaling, and mirroring for the no-complexity navigation scenes in the datasets to expand this part of the data sample by a factor of 5. However, the complexity perception results of the navigation scenes were not significantly improved by reclustering and computing the expanded datasets. The reason for this phenomenon may be due to the GLCM and Tamura coarseness methods employed in the texture extraction process, which are insensitive to rotation, translation, scaling, and mirroring operations. This is because the above operations only linearly transform the image and do not substantially change the distribution of textures in the navigation scenes. This phenomenon further demonstrates that our proposed textural feature parameters can be used to perceive and distinguish navigation scenes with different levels of complexity.

## Conclusions

This paper constructs a USV navigation scene complexity perception system based on image texture features. Here, the level of complexity classification and accurate perception calculation method are obtained by modeling and analyzing the navigation scene complexity problem. The first contribution of this paper is that it sorts out the multiple elements contained in complex scenes to cover as many test scenes as possible, solves the long-tail problem, and summarizes a total of five typical elements of cluttered background, dynamic obstacles, camera shaking, cast shadows, and special weather conditions. Then, four levels of complexity are divided according to whether the current navigation scene contains the above typical complex scene elements and their quantity. These levels are no complexity $L_N$, low complexity $L_L$, medium complexity $L_M$ and high complexity $L_H$. The other main contribution is that the work proposes a scene complexity perception method based on image texture features. The method first uses the GLCM and Tamura coarseness to obtain a 5-dimensional texture feature vector. Then, a novel PBBPSC algorithm is designed to cluster the scene complexity, and based on the classification results, the interval mapping method is applied to obtain the exact value of the navigation scene complexity. Finally, it is proven that the method in this paper can not only describe the level of complexity of navigation scenes but also obtain accurate complexity values, which are verified using classical and self-collected datasets. Next, based on the obtained navigation scene complexity, we will further study and improve the navigation scene testing system to provide support for the development of USV testing technology.

## Data Availability

The datasets generated during and/or analyzed during the current study are available from the corresponding author on reasonable request.
